# SVIP in plasma: a candidate blood-based biomarker for early detection of amnestic mild cognitive impairment

**DOI:** 10.3389/fnagi.2026.1781331

**Published:** 2026-05-29

**Authors:** Gaigai Lu, Hui Shan, Yuxin Yin, Lele Chen, Keyan Yu, Zhuonan Wei, Hui Chen, Lin Hu, Yulong Qi, Tong Wu, Silin Tao, Xiang Fan, Guanxun Cheng

**Affiliations:** 1 Department of Medical Imaging, Peking University Shenzhen Hospital, Shenzhen, China; 2 Institute of Precision Medicine, Peking University Shenzhen Hospital, Shenzhen, China

**Keywords:** Alzheimer’s disease, blood-based biomarkers, deep plasma proteomics, mild cognitive impairment, SVIP, VCP

## Abstract

**Background:**

Amnestic mild cognitive impairment (aMCI) represents a critical clinical window for early intervention in Alzheimer’s disease (AD). Identifying readily detectable, high-abundance plasma biomarkers for aMCI remains clinically important. Overexpression of Valosin-Containing Protein (VCP) has been shown to enhance autophagy and reduce tau levels in AD animal models. Notably, VCP, with a molecular weight of 90 kDa, typically assembles into hexamers, which is hypothesized to restrict its ability to cross the blood–brain barrier even under neurodegenerative conditions. In contrast, the small VCP-interacting protein (SVIP), with a molecular weight of only 9 kDa, interacts with VCP to maintain the dynamic stability of autophagosomes within cells. Therefore, we aim to explore plasma SVIP as a peripheral blood biomarker for aMCI and assess whether it can outperform VCP in detecting aMCI.

**Methods:**

This was a retrospective study based on the STAR (Shenzhen Multi-modal Aging Research) cohort. Participants were recruited as a convenience sample. Eighty-four participants (44 cognitively unimpaired [CU], 40 aMCI) were included, with diagnostic classification based on standardized clinical and neuropsychological criteria. Plasma levels of SVIP and VCP were measured using deep plasma proteomics enriched with biofunctional magnetic beads. Diagnostic performance was evaluated using Receiver Operating Characteristic (ROC) analysis and DeLong’s test. This study was registered in the Chinese Clinical Trial Registry (ChiCTR2200066700).

**Results:**

Compared to the CU group, the aMCI group showed significantly decreased SVIP (*p* < 0.001), while no significant difference was observed in VCP levels (*p* = 0.823). The area under the curve (AUC) of SVIP in detecting aMCI was 0.836 (95% CI: 0.739 to 0.908), significantly higher than VCP [AUC = 0.513 (95% CI: 0.401 to 0.624)] (*p* < 0.0001, DeLong’s test).

**Conclusion:**

Plasma SVIP demonstrates significantly higher diagnostic accuracy than VCP for the detection of aMCI, suggesting its potential as a candidate blood-based biomarker pending large-scale pathophysiological validation.

## 1 Introduction

Alzheimer’s disease (AD) is the most prevalent form of dementia, accounting for 60 to 70% of all cases. It is characterized by progressive memory loss, cognitive decline, and behavioral disturbances, which significantly impair the quality of life in elderly individuals (Alzheimer’s Association, 2024). The onset of AD is typically insidious, with a gradual and progressive course, making early diagnosis challenging and placing a substantial burden on patients, their families, and healthcare systems. Mild cognitive impairment (MCI) represents an intermediate clinical stage between cognitively unimpaired (CU) and dementia, of which amnestic MCI (aMCI) is the most common type of MCI, with a high risk of progression to AD (Knopman et al., 2021). However, not all individuals with aMCI will inevitably develop AD—approximately 14.9% may experience cognitive improvement with timely intervention (Salemme et al., 2025). Therefore, early identification of aMCI is crucial for delaying disease progression and improving patient outcomes.

According to the 2024 National Institute on Aging and the Alzheimer’s Association (NIA-AA) diagnostic framework, the diagnosis of Alzheimer’s disease (AD) is primarily based on detecting biomarkers in cerebrospinal fluid (CSF), blood, and positron emission tomography (PET) imaging (Jack et al., 2024). However, these approaches present significant limitations in clinical practice. CSF collection is inherently invasive, often resulting in low patient compliance. PET imaging is constrained by cost and radiation exposure (Ren et al., 2022; Hansson, 2021). Recently, peripheral blood biomarkers—being less invasive and easily accessible—have shown great promise for detecting AD, especially at the aMCI stage (Palmqvist et al., 2025). However, existing plasma biomarkers such as p-tau217 are available at extremely low plasma concentrations, down to sub-picogram/mL levels. Even on the highly sensitive Simoa platform, certain p-tau isoform concentrations may be below the lower limit of quantification. Moreover, Simoa assays are costly and subject to batch-to-batch variability. These constraints limit their availability to specialized centers, hindering widespread adoption for large-scale screening (Hu et al., 2022; Hampel et al., 2023; Leuzy et al., 2022). Therefore, the development of novel plasma biomarkers that are cost-effective, highly sensitive, and widely accessible is urgently needed to enable early screening and diagnosis of aMCI.

The etiology of AD is complex, and the specific pathogenesis has not been fully clarified. Emerging evidence suggests that autophagy dysfunction may play a pivotal role in the early stages of AD, potentially preceding deposition (Lee, 2022; Palmer et al., 2025). This raises the possibility that autophagy dysfunction might contribute to neurodegeneration at an early stage and could potentially be detectable before the formation of Aβ plaques. Recent studies have shown that VCP plays an important regulatory role in the autophagy-related pathology of AD. *In vivo*, in zebrafish and mouse models, overexpression of VCP significantly reduced phosphorylated and oligomerized/aggregated tau levels by promoting autophagic flux and improved cognitive-behavioral phenotypes induced by tau. Clinicopathological studies have further confirmed that the expression of VCP is significantly downregulated in the brains of patients with AD, which is consistent with its role in tau clearance (Darwich et al., 2020; Giong et al., 2024). However, to our knowledge, studies investigating the diagnostic value of VCP in peripheral blood remain limited. Notably, VCP has a molecular weight of approximately 90 kDa and usually presents in the form of hexamers, which may limit its penetration into the peripheral circulation even under conditions of blood–brain barrier (BBB) breakdown (Chu, 2023). In contrast, SVIP, a specific cofactor of VCP with a molecular weight of only 9 kDa, functions by recruiting VCP to lysosomes and modulating the stability of intracellular autophagic vesicles (Johnson et al., 2021; Akcan et al., 2020; Wang et al., 2011). SVIP’s low molecular weight (9 kDa) falls within the theoretical range (~10 kDa) during BBB leakage (Haruwaka et al., 2019), potentially enhancing its detectability in plasma.

This study aimed to evaluate the diagnostic efficacy of SVIP as a peripheral blood biomarker for the detection of aMCI and to determine whether plasma SVIP demonstrates superior diagnostic performance compared to VCP.

## 2 Methods

### 2.1 Participants

This was a retrospective diagnostic accuracy study based on the STAR (Shenzhen Multi-modal Aging Research) cohort. From December 2023 to March 2024, participants in this study were recruited through poster advertisements and the Memory Clinic at Peking University Shenzhen Hospital, using a convenience sampling strategy. A total of 84 participants were enrolled and divided into the aMCI group (*n* = 40) and the CU group (*n* = 44) based on clinical diagnostic criteria. The detailed participant selection process is illustrated in Supplementary Figure S1. All participants were of Han Chinese ethnicity. Baseline data were collected for all participants, including demographic information, clinical variables, neuropsychological assessments, and plasma biomarker measurements. Neuropsychological assessments and blood sample collection were performed on the same day for most participants, while a small subset completed blood sampling within two weeks after cognitive assessment. This study was conducted following the Declaration of Helsinki and approved by the Ethics Committee of Peking University Hospital (No. 2022–160-01). Written informed consent was obtained from each participant before their inclusion in the study. This trial was registered with the Chinese Clinical Trial Registry (Identifier: ChiCTR2200066700) on December 14, 2022.

Diagnostic classification was determined through comprehensive clinical evaluation in conjunction with standardized neuropsychological assessments. The neuropsychological battery included the Mini-Mental State Examination (MMSE), Montreal Cognitive Assessment (MoCA), Auditory Verbal Learning Test (AVLT), Hamilton Anxiety Rating Scale (HAMA), the 15-item Geriatric Depression Scale (GDS-15), and Activities of Daily Living (ADL), which were used to comprehensively evaluate global cognition, episodic memory, and affective status. For the CU group, inclusion criteria were: (1) age between 55 and 80 years; (2) negative plasma p-tau217 status (Cognitive unimpaired participants with preclinical AD were excluded according to the 2024 revised diagnostic and staging framework for AD (Jack et al., 2024)) (3) a Clinical Dementia Rating (CDR) score of 0; (4) no objective memory impairment on neuropsychological testing and (5) preserved ADLs with full functional independence. For the aMCI group, diagnosis primarily followed the established clinical criteria for aMCI (Albert et al., 2011; Petersen, 2004) and met Clinical Stage 3 of the 2024 revised diagnostic and staging framework (Jack et al., 2024). The inclusion criteria were as follows: (1) age between 55 and 80 years; (2) memory complaint; (3) a CDR score of 0.5; (4) objective memory impairment on standardized neuropsychological tests, defined as performance ≥1.5 standard deviations below age- and education-adjusted normative values and (5) preserved independence in ADLs, with possible mild inefficiency in complex ADLs. No formal classification into single-domain or multidomain aMCI was performed in this study. All aMCI participants were defined by predominant episodic memory impairment. Participants with non-amnestic MCI were excluded during screening to ensure a homogeneous aMCI cohort. Participants with cognitive impairment due to other causes, such as vascular cognitive impairment, disorders of consciousness, epilepsy, or psychiatric illnesses, were excluded. Participants receiving anti-dementia medications, including cholinesterase inhibitors (e.g., donepezil) or other Alzheimer’s disease–related treatments, were excluded to avoid potential pharmacological effects on cognitive performance. Additionally, individuals unable to undergo MRI examinations or complete neuropsychological evaluations due to physiological limitations or sensory impairments (e.g., visual or hearing impairments) were also excluded from the study. MRI examinations were performed as part of the screening procedure to exclude structural brain abnormalities, including cerebrovascular lesions, tumors, and other neurological conditions that may affect cognitive function. Detailed classification of exclusion criteria is provided in Supplementary Table S1.

### 2.2 Blood processing and plasma storage

Whole blood samples were collected via elbow vein puncture using a vacuum blood collection needle for 5 mL into polypropylene blood collection tubes containing K2-EDTA anticoagulant. The tubes were immediately subjected to gentle inversion several times following collection. Within 30 min of collection, whole blood samples were centrifuged in a pre-cooled centrifuge at 3,000 × g for 10 min at 4 °C. Subsequently, the plasma layer was separated and dispensed into 500-μL aliquots using a sterile pipette. All whole blood samples were processed within 2 h of collection and stored at −80 °C. The number of freeze–thaw cycles was strictly limited to no more than one to ensure sample integrity and stability.

### 2.3 Analysis of blood biomarkers based on deep proteomics technology

#### 2.3.1 Deep plasma proteomics sample preparation

The sample preparation process included enrichment of low-abundance proteins, protein denaturation, reduction and alkylation, enzymatic hydrolysis, and peptide purification. The procedures were as follows: A total of 1 mg of nanoparticle magnetic beads (OSFP0002 kit, Shanghai Omicron Biotechnology Co., Ltd.) was mixed with 100 μL of washing buffer. Subsequently, 100 μL of plasma was added to the mixture and incubated at 37 °C with shaking at 1,000 rpm for 1 h. Following incubation, the beads were collected using a magnetic separation device and washed three times with 300 μL of washing buffer. The precipitate was resuspended with 40 μL of lysis buffer and heated at 95 °C, 1000 rpm with shaking for 5 min. After cooling to room temperature, Lys-C and Trypsin enzymatic hydrolysate were added successively. The enzymatic hydrolysate was oscillated at 37 ° C and 1,000 rpm for 2 h. The reaction was terminated with 10% trifluoroacetic acid. Desalination and purification were performed using the C18 peptide purification column. The peptides were eluted twice with 30 μL of eluent, and finally dried and stored using a vacuum centrifugal concentrator.

#### 2.3.2 DIA LC–MS/MS analysis

Nanoliter liquid chromatography–tandem mass spectrometry (nano-LC–MS/MS) analysis was performed on an Orbitrap Exploris 480 mass spectrometer (Thermo Fisher Scientific) equipped with the Nanoscale ultra-high performance liquid chromatography system (Thermo Scientific Ultimate 3,000). The peptide fragments were first enriched on a capture column (Acclaim™ PepMap™ 100 C18 HPLC, 75 μm × 2 cm, Thermo Scientific) and subsequently separated on the analysis column (Acclaim™ PepMap™ 100 C18 HPLC, 75 μm × 25 cm, Thermo Scientific). The loading volume was 1 μg, and the mobile phase was an acetonitrile (ACN) solution containing 0.1% formic acid (FA). Gradient elution (3–90% ACN) was performed at a flow rate of 300 nL/min for 120 min. Both the precursor and fragment ions were collected in the Orbitrap mass analyzer with the following key parameter settings: radiofrequency (RF) lens frequency of 40%, spray voltage of 2.5 kV, and ion transfer tube temperature of 320 °C. The full-scan MS range was set at 350–1,200 m/z, with a resolution of 120,000 (AGC target value of 1 × 10^6, maximum injection time of 50 ms).

#### 2.3.3 Data processing and quantitative analysis

DIA raw data were processed using Spectronaut version 17 (Biognosys, Switzerland), a vendor-independent software platform specifically designed for DIA-based quantitative proteomics. Protein identification was performed by searching against the UniProtKB *Homo sapiens* reference proteome (taxonomy ID: 9606; Release 2023_05). The false discovery rate (FDR) was controlled at 1% at the peptide level using a target–decoy strategy implemented in Spectronaut. Protein quantification was based on unique peptides. SVIP was quantified using 2 unique peptides. VCP was quantified using multiple peptides, with the number of identified peptides per sample ranging from 40 to 88, ensuring robust and reliable protein-level quantification. Signal normalization was performed using the default local normalization strategy implemented in Spectronaut (automatic normalization mode), which corrects for systematic technical variation across runs. To evaluate measurement stability, coefficients of variation (CVs) across samples were calculated. The CV for SVIP was approximately 60%, reflecting inter-individual biological variability in plasma levels. The CV for VCP was approximately 23%, indicating relatively stable quantitative performance. Both SVIP and VCP were successfully quantified in all 84 plasma samples, resulting in a missingness rate of 0%. The quantified protein levels were derived from LC–MS signal intensities and were therefore expressed as relative abundances in arbitrary units (a.u.). All biomarker measurements were performed blinded to clinical group assignment.

### 2.4 Statistical analysis

Sample size was estimated using PASS software (version 25.0.2), assuming a two-sided *α* of 0.05 and a statistical power of 0.80 with equal group allocation. The minimum required sample size was 13 participants per group, which was increased to 17 per group after accounting for a 20% attrition rate. All statistical analyses were performed using SPSS version 25.0. The normality of continuous variables was assessed separately for each group using the Shapiro–Wilk test. Visual inspection of histograms and Q-Q plots was also performed. Variables that were not normally distributed in either group were described as median (interquartile range, IQR) and analyzed with non-parametric Mann–Whitney U tests. Plasma VCP levels, which were normally distributed, were described as Means (Standard Deviation) and analyzed using independent-sample t-tests. Chi-square tests were used for categorical variable comparisons between groups. Receiver operating characteristic (ROC) curves were constructed to evaluate the diagnostic performance of VCP and SVIP for discriminating aMCI from CU participants. Multivariable-adjusted ROC models were generated by adjusting for age and years of education. Calibration curves were generated to assess the agreement between predicted probabilities and observed outcomes. The Hosmer–Lemeshow goodness-of-fit test and Brier score were used to evaluate model calibration and overall predictive performance, respectively. The area under the curve (AUC), along with the corresponding 95% confidence intervals (CIs), was calculated. In addition, optimal cutoff values were determined using the Youden index, and diagnostic performance metrics, including sensitivity, specificity, positive predictive value (PPV), and negative predictive value (NPV), were calculated. Differences between AUCs were examined using the DeLong test. Spearman’s rank correlation analysis was used to assess correlations between plasma biomarkers (SVIP and VCP) and cognitive scores (MMSE and MoCA). Multivariable linear regression analyses were performed to evaluate adjusted associations, controlling for age and years of education. All statistical graphs, including boxplots (for group comparisons) and scatter plots (for correlation analysis), were created using GraphPad Prism version 10.4.0. ROC curves were plotted using MedCalc version 22.0. Confusion matrix plots and calibration curves were generated using Python 3.13.5. A two-tailed *p* value of < 0.05 was considered statistically significant.

## 3 Results

### 3.1 Study population characteristics

From December 2023 to March 2024, a total of 84 subjects from the STAR cohort were included in the final analysis, including 44 cases in the CU group and 40 cases in the aMCI group. All participants were of Han Chinese ethnicity. Group comparisons revealed significant differences between the CU and aMCI groups in age, years of education, smoking status, MMSE scores, MoCA scores, and all AVLT subscales (*p* < 0.05). Compared with the CU group, the aMCI group showed significantly lower MMSE, MoCA, and AVLT performance. No significant differences were observed in sex, comorbidities (including hypertension, diabetes, and hyperlipidemia), alcohol consumption, ADL, GDS-15, or HAMA scores between the two groups (*p* > 0.05). Detailed demographic and cognitive assessment data are summarized in Table 1.

**Table 1:** Baseline characteristics of participants in the CU and aMCI groups.

Characteristics	CU (*n* = 44)	aMCI (*n* = 40)	*p*
Demographics			
Age, years, median (IQR)	58.50 (6.00)	65.00 (11.00)	<0.001^*^
Female, *n* (%)	31 (70.5)	29 (72.5)	0.836
Education, years, median (IQR)	14.00 (4.10)	9.50 (6.40)	<0.001^*^
Comorbidities			
Hypertension, *n* (%)	9 (20.5)	16 (40.0)	0.050
Diabetes, *n* (%)	1 (2.3)	5 (12.8)	0.153
Dyslipidemia, *n* (%)	15 (34.1)	14 (35.0)	0.930
Lifestyle			
Smoking, *n* (%)	0 (0)	6 (15.0)	0.025^*^
Alcohol consumption, *n* (%)	8 (18.2)	5 (12.5)	0.472
Neuropsychological scale scores			
MMSE, median (IQR)	29.00 (2.00)	27.00 (2.00)	<0.001^*^
MoCA, median (IQR)	27.00 (2.00)	20.00 (4.00)	<0.001^*^
ADL, median (IQR)	20.00 (0.00)	20.00 (1.00)	0.158
AVLT - 1, median (IQR)	6.00 (3.00)	5.00 (2.00)	0.037^*^
AVLT - 2, median (IQR)	9.00 (2.00)	7.00 (4.00)	<0.001^*^
AVLT - 3, median (IQR)	11.00 (3.00)	8.50 (4.00)	<0.001^*^
AVLT - Delayed, median (IQR)	11.00 (3.00)	7.00 (5.00)	<0.001^*^
AVLT - Recognition, median (IQR)	13.00 (3.00)	10.00 (5.00)	<0.001^*^
GDS -15 score, median (IQR)	2.00 (2.00)	2.00 (5.00)	0.064
HAMA, median (IQR)	2.00 (3.00)	2.50 (6.00)	0.220
Blood biomarkers			
SVIP (×10^5^ a.u.), median (IQR)	3.46 (2.17)	1.53 (0.88)	<0.001^*^
VCP (×10^6^ a.u.), mean ± SD	1.05 ± 0.24	1.06 ± 0.26	0.823

Between-group comparisons were performed using Student’s t-test, Mann–Whitney U test, Chi-square test, or Fisher’s exact test as appropriate. CU, cognitively unimpaired; aMCI, amnestic mild cognitive impairment; MMSE, Mini-Mental State Examination; MoCA, Montreal Cognitive Assessment; ADL, Activities of Daily Living; AVLT, Auditory Verbal Learning Test; GDS-15: 15-item Geriatric Depression Scale; HAMA, Hamilton Anxiety Rating Scale; SVIP, small VCP-interacting protein; VCP, valosin-containing protein; a.u., arbitrary units; IQR, interquartile range; SD, standard deviation. *p < 0.05.

### 3.2 SVIP levels are significantly decreased in aMCI, while VCP levels show no remarkable difference

**Figure 1:**
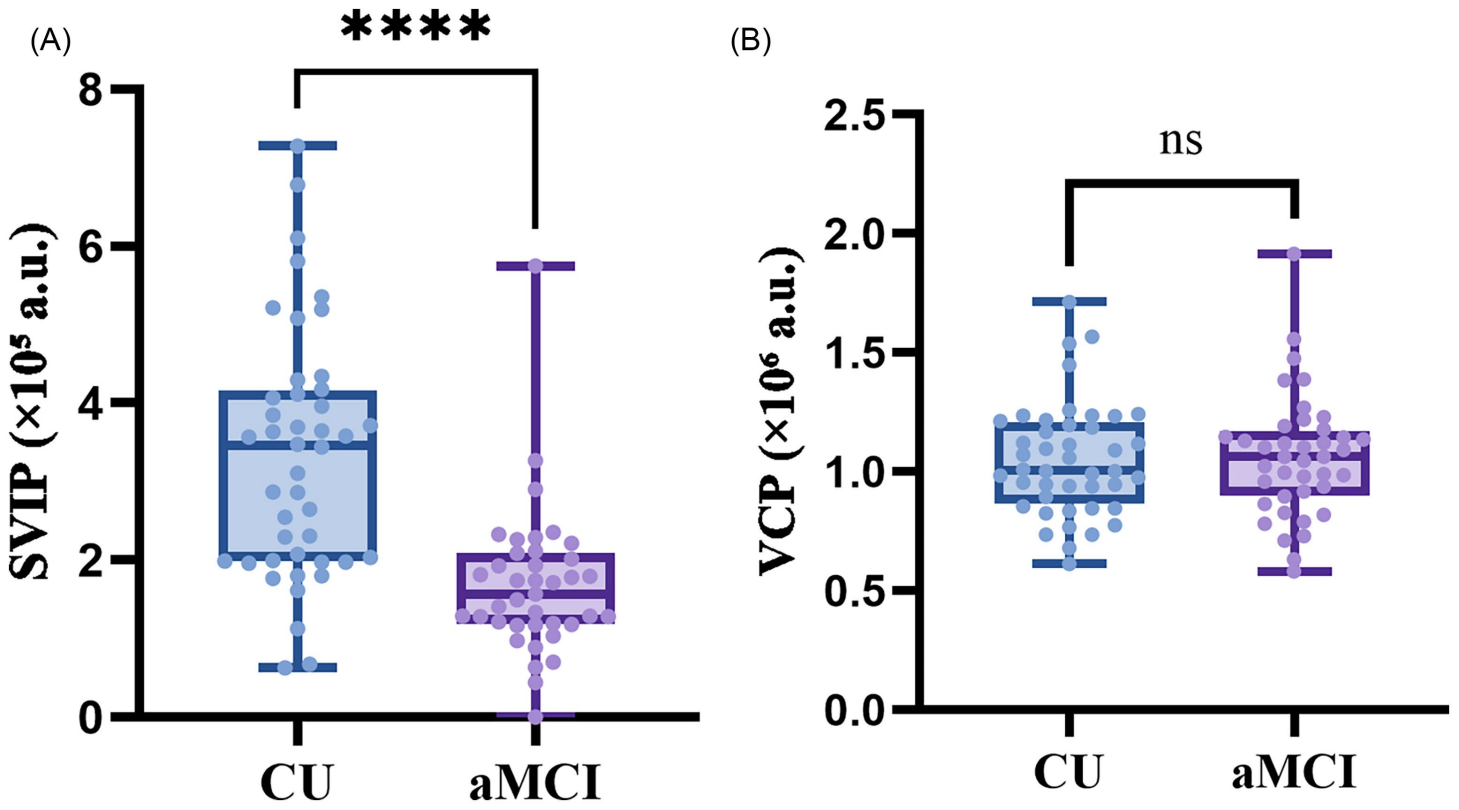
Comparison of SVIP and VCP levels between CU and aMCI groups. **(A)** SVIP levels in CU and aMCI groups. **(B)** VCP levels in CU and aMCI groups. CU, cognitively unimpaired; aMCI, amnestic mild cognitive impairment; SVIP, small VCP-interacting protein; VCP, valosin-containing protein. ****, *p* < 0.0001; ns, not significant.

As outlined in the Methods section, plasma levels of SVIP and VCP were measured using deep proteomics technology. The results demonstrated a significant reduction in the median plasma level of SVIP in the aMCI group compared to the CU group (aMCI: 1.53 [IQR: 0.88] vs. CU: 3.46 [IQR: 2.17], *p* < 0.001). In contrast, there was no statistically significant difference in plasma VCP levels between the two groups (aMCI: 1.06 ± 0.26 vs. CU: 1.05 ± 0.24, *p* = 0.823). These findings are visually presented in Figure 1.

### 3.3 Plasma SVIP demonstrates high diagnostic accuracy for aMCI

**Figure 2:**
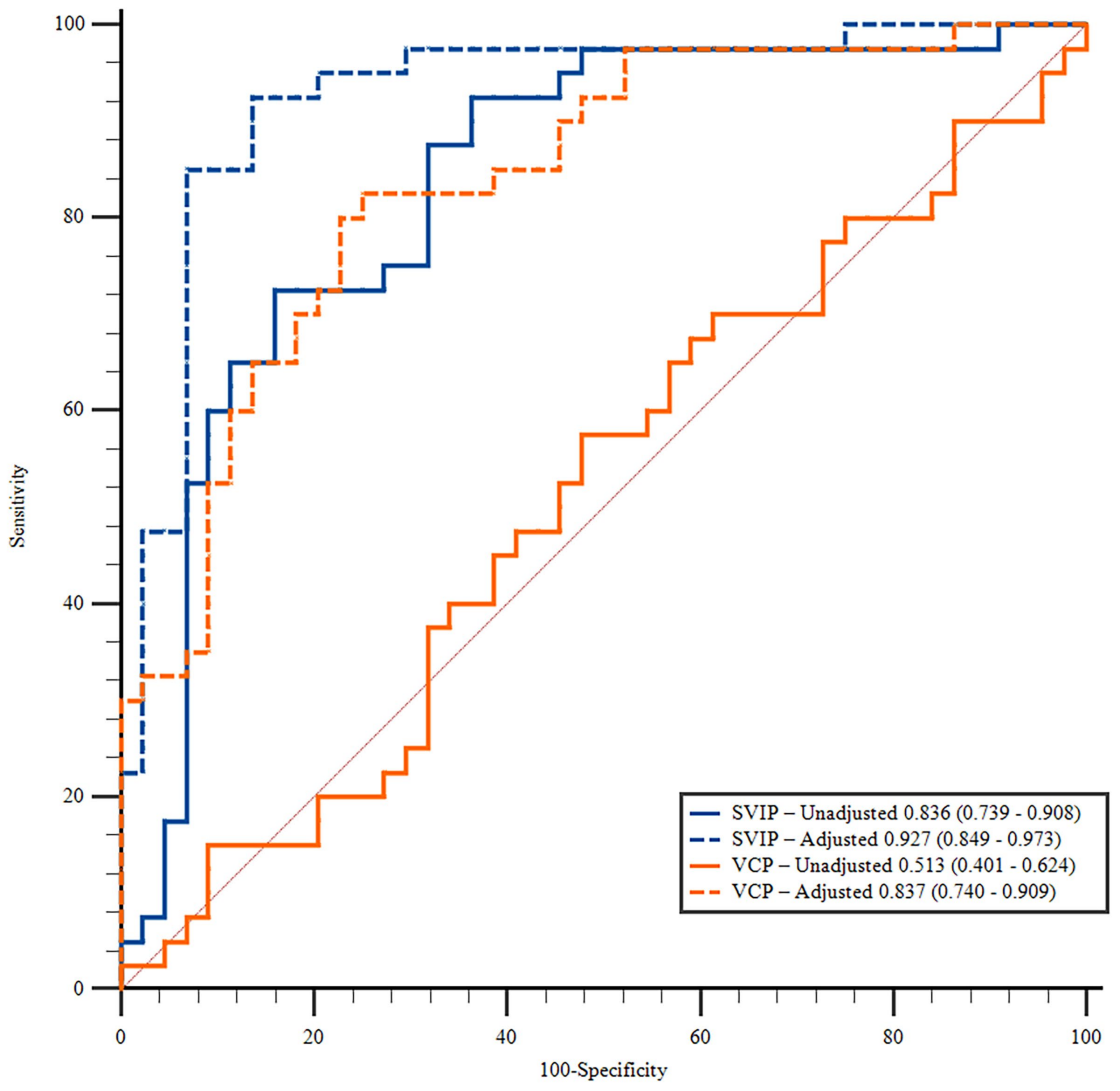
ROC curves of SVIP and VCP for discriminating aMCI from CU participants. Unadjusted and adjusted models (adjusted for age and years of education) are presented. AUC values with 95% confidence intervals are reported in the figure. CU, cognitively unimpaired; aMCI, amnestic mild cognitive impairment; SVIP, small VCP-interacting protein; VCP, valosin-containing protein.

**Figure 3:**
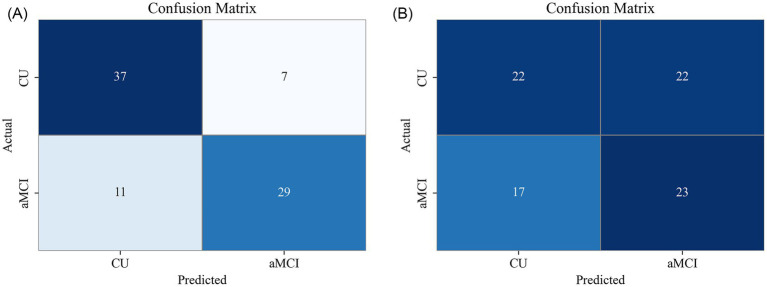
Confusion matrix analysis of SVIP **(A)** and VCP **(B)** for classification of aMCI and CU participants. Classification was based on optimal cutoff values determined by the Youden index from ROC curve analysis. CU, cognitively unimpaired; aMCI, amnestic mild cognitive impairment; SVIP, small VCP-interacting protein; VCP, valosin-containing protein.

ROC curve analysis was conducted to assess the diagnostic efficacy of plasma SVIP and VCP in differentiating aMCI from CU. The results showed that SVIP exhibited excellent diagnostic performance, with an AUC of 0.836 (95% CI: 0.739 to 0.908). Using an optimal cutoff of 1.935, SVIP achieved a sensitivity of 72.5% and a specificity of 84.1%, with a PPV of 80.6% and an NPV of 77.1%. In contrast, VCP showed poor diagnostic utility, with an AUC of only 0.513 (95% CI: 0.401 to 0.624). Using a cutoff of 1.010, VCP yielded a sensitivity of 57.5% and a specificity of 52.3%, with a PPV of 51.1% and an NPV of 56.4%. The superiority of SVIP over VCP was further confirmed by the DeLong test (*p* < 0.0001). These results are presented in Figure 2. Confusion matrix analyses based on the optimal cutoff values are presented in Figure 3, and detailed diagnostic performance metrics are summarized in Table 2.

**Table 2:** Diagnostic performance of SVIP and VCP for differentiating aMCI from CU based on ROC analysis.

Blood biomarkers	AUC (±SE)	95% CI	Cut-off value	Specificity%	Sensitivity%	PPV%	NPV%
SVIP (×10^5^ a.u.)	0.836 ± 0.046	0.739–0.908	<1.935	84.1	72.5	80.6	77.1
VCP (×10^6^ a.u.)	0.513 ± 0.064	0.401–0.624	>1.010	52.3	57.5	51.1	56.4

ROC, receiver operating characteristic; AUC, area under the curve; SE, standard error; CI, confidence interval; PPV, positive predictive value; NPV, negative predictive value. Optimal cutoff values were determined using the Youden index. Sensitivity, specificity, PPV, and NPV were calculated based on the optimal cutoff values.

**Figure 4:**
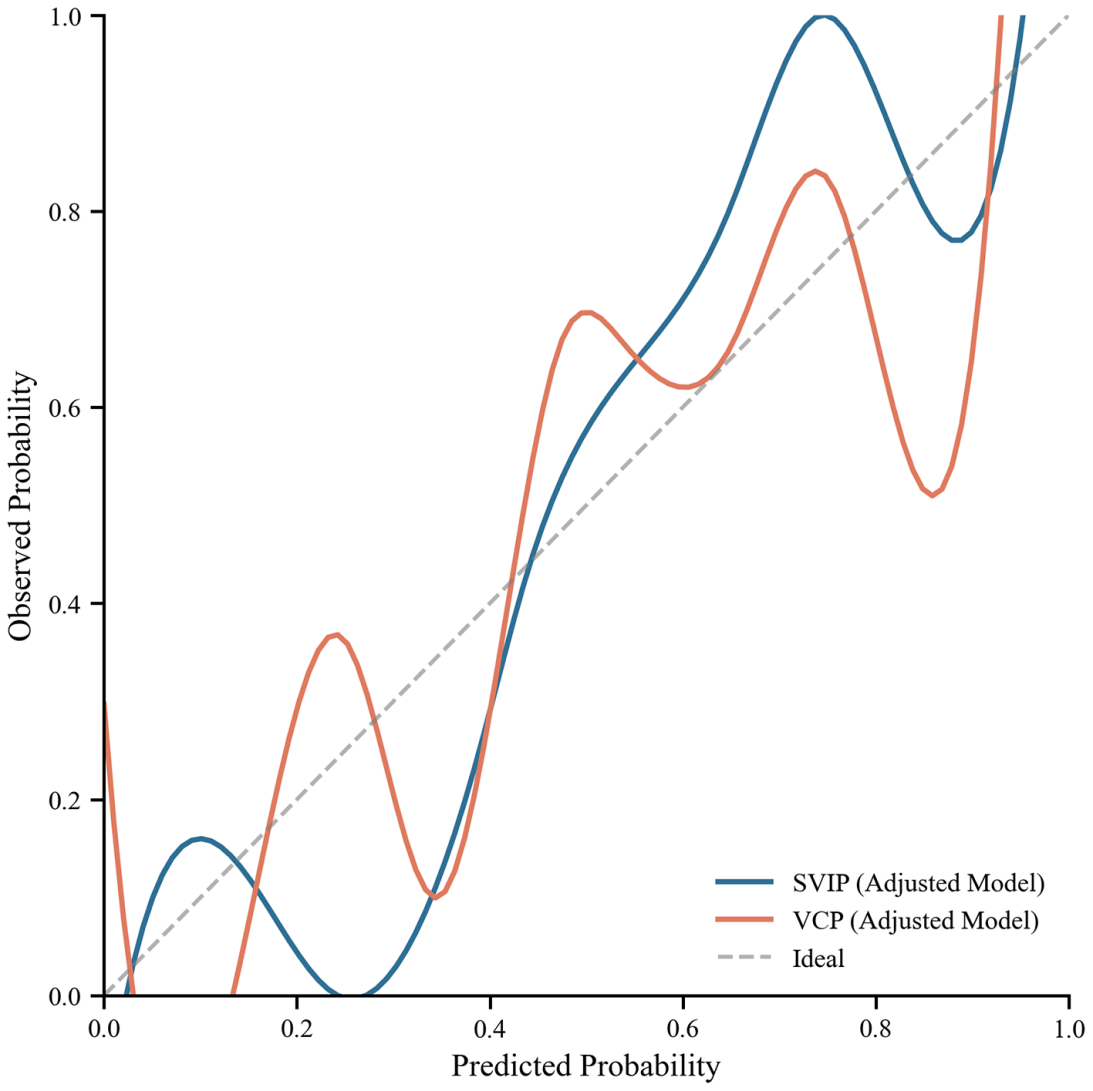
Calibration plots of adjusted SVIP- and VCP-based models for prediction of aMCI. The models were adjusted for age and years of education. The ideal line represents perfect calibration. Calibration performance was assessed using the Hosmer–Lemeshow goodness-of-fit test, and overall predictive performance was evaluated using the Brier score. CU, cognitively unimpaired; aMCI, amnestic mild cognitive impairment; SVIP, small VCP-interacting protein; VCP, valosin-containing protein.

After adjustment for age and years of education, the diagnostic performance of SVIP improved, with an AUC of 0.927 (95% CI: 0.849 to 0.973), and that of VCP also increased, with an AUC of 0.837 (95% CI: 0.740 to 0.909). DeLong’s test showed a significant difference between SVIP and VCP after adjustment (*p* = 0.020). The corresponding adjusted ROC curves are shown in Figure 2. Calibration analysis showed acceptable agreement between predicted probabilities and observed outcomes for both adjusted models (Hosmer–Lemeshow test: SVIP, *p* = 0.504; VCP, *p* = 0.746). The adjusted SVIP-based model exhibited a lower Brier score than the adjusted VCP-based model (0.102 vs. 0.171), indicating better calibration accuracy (Figure 4).

### 3.4 Plasma SVIP levels are positively correlated with cognitive performance in aMCI

**Figure 5:**
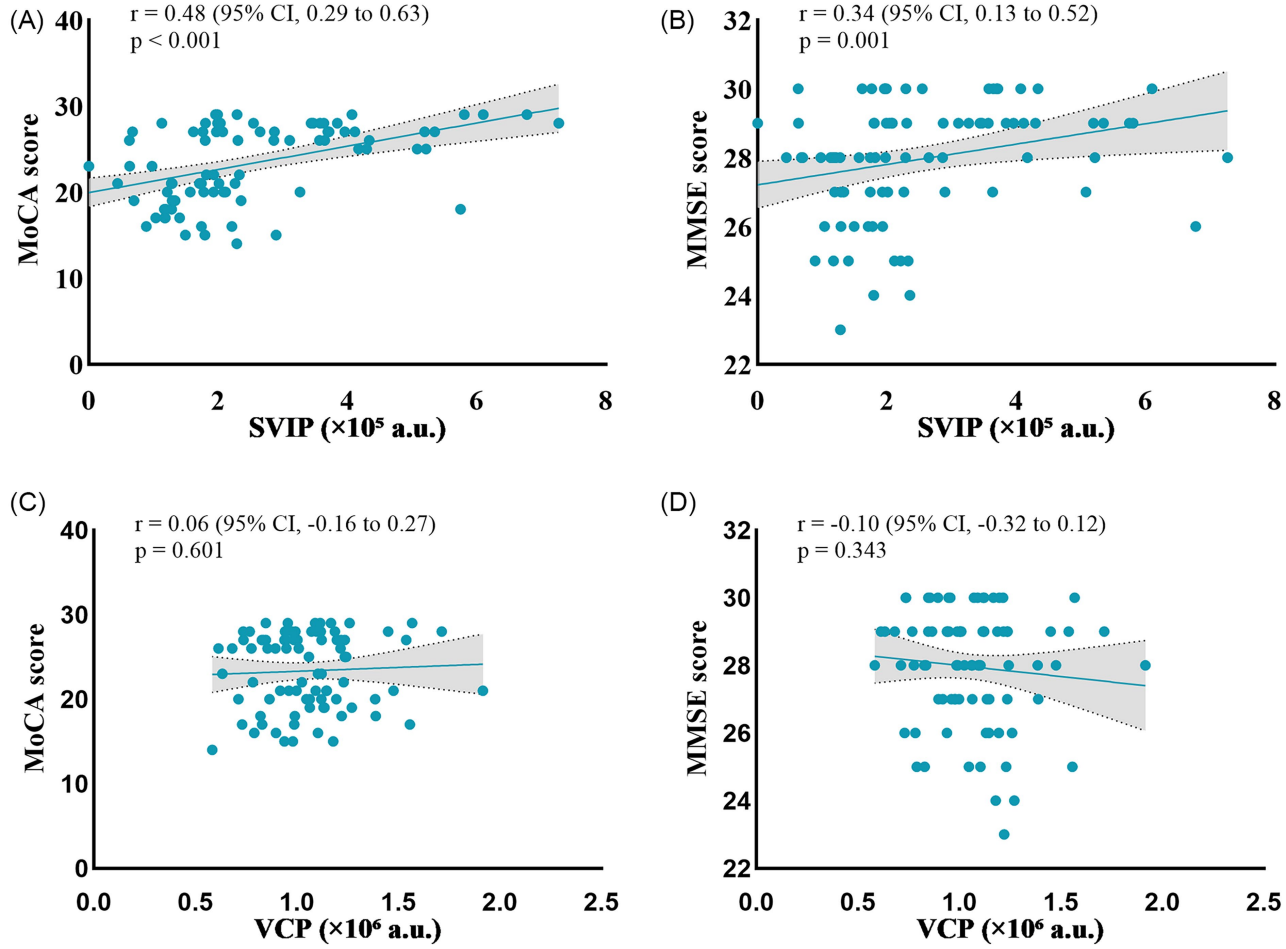
Correlations between plasma biomarkers and cognitive performance. Scatter plots show the associations between SVIP and MoCA **(A)** and MMSE **(B)**, and between VCP and MoCA **(C)** and MMSE **(D)**. Solid lines represent linear regression fits, with shaded areas indicating 95% confidence intervals. Correlation coefficients (*r*) and corresponding *p* values are shown in each panel. CU, cognitively unimpaired; aMCI, amnestic mild cognitive impairment; SVIP, small VCP-interacting protein; VCP, valosin-containing protein; MMSE, Mini-Mental State Examination; MoCA, Montreal Cognitive Assessment.

We analyzed the correlations between plasma SVIP and VCP levels and neuropsychological assessments, including MMSE and MoCA scores. Significant positive correlations were observed between SVIP levels and MMSE scores (r = 0.34, 95% CI, 0.13 to 0.52, *p* = 0.001), as well as between SVIP levels and MoCA scores (r = 0.48, 95% CI, 0.29 to 0.63, *p* < 0.001). In contrast, VCP levels showed no significant correlations with MMSE (r = −0.10, 95% CI, −0.32 to 0.12, *p* = 0.343) or MoCA scores (r = 0.06, 95% CI, −0.16 to 0.27, *p* = 0.601). These results are illustrated in Figure 5.

To further control for potential confounding effects of age and years of education, multivariable linear regression analyses were performed. SVIP was independently associated with MoCA scores (*β* = 0.98, 95% CI, 0.53 to 1.42, *p* < 0.001), whereas no significant association was observed with MMSE scores (β = 0.18, 95% CI, −0.03 to 0.38, *p* = 0.094). In contrast, VCP showed no significant associations with either MMSE (β = −0.58, 95% CI, −1.89 to 0.73, *p* = 0.381) or MoCA scores (β = 1.95, 95% CI, −1.13 to 5.03, *p* = 0.212). These results, along with the regression coefficients for age and years of education, are presented in Supplementary Table S2.

## 4 Discussion

In this study, we explored the potential of two proteins—SVIP and VCP, which have been implicated in cellular processes relevant to neurodegeneration—as candidate plasma biomarkers associated with phenotypic aMCI. Our results showed that plasma SVIP levels were significantly reduced in individuals with phenotypic aMCI and exhibited good diagnostic performance (AUC = 0.836, *p* < 0.001). However, its clinical specificity warrants further validation against established core AD biomarkers. In contrast, no significant differences were observed in plasma VCP levels at this stage.

Our findings showed a significant reduction in plasma SVIP levels during the phenotypic aMCI stage. It should be noted that aMCI in this cohort was defined based on clinical evaluation and standardized neuropsychological assessments, without confirmation via AD-specific biomarkers such as amyloid PET or CSF assays. Future studies are needed to validate these findings in biomarker-defined cohorts. This clinical observation is consistent with mechanisms proposed by earlier studies. SVIP, a regulatory protein that binds to VCP, is expressed in the cerebral cortex and cerebellum, suggesting a potential role in maintaining neuronal homeostasis (Wang et al., 2011). At the cellular level, SVIP and VCP co-localize in the neuronal perikaryon, where SVIP facilitates the recruitment of VCP to the lysosomal membrane (Johnson et al., 2021; Wu et al., 2013). This localization may be functionally important, as it enhances the interaction between VCP and autophagy-related proteins, including LC3-II, p62, and LAMP1, which have been reported to promote autophagosome-lysosome fusion and sustain autophagic flux (Gill et al., 2024). Conversely, SVIP deficiency has been shown to disrupt lysosomal network integrity and block autophagic flux, leading to abnormal protein accumulation and neurodegenerative changes (Johnson et al., 2021). VCP has been implicated in autophagy-related degradation of pathological tau in experimental models (Hill, 2021; Wang et al., 2024; Wrobel et al., 2021). Given that SVIP directly regulates VCP function, this interaction may represent a potential biological pathway underlying its biological effects. Beyond its role in autophagy, SVIP also appears to function as a bifunctional regulator of protein quality control. By competitively binding to the N-terminal domain of VCP, SVIP negatively regulates the endoplasmic reticulum-associated degradation (ERAD) pathway, which may influence ubiquitination and degradation of misfolded proteins (Ballar et al., 2007; Liu et al., 2013). Notably, excessive activation of ERAD may exacerbate endoplasmic reticulum stress and cellular damage, while SVIP-mediated suppression of ERAD could theoretically contribute to a compensatory protective mechanism.

Although our study showed a significant decrease in plasma SVIP levels in phenotypic aMCI, this finding appears inconsistent with previous reports based on post-mortem brain tissue-derived extracellular vesicles, which observed elevated SVIP levels associated with MCI (You et al., 2022). This discrepancy may be attributed to several factors. First, post-mortem extracellular vesicle analyses and peripheral blood-based analyses reflect different biological compartments and may not fully capture systemic influences, including contributions from peripheral tissues such as the liver and endothelial cells (Vandendriessche et al., 2022), which may introduce systematic bias in the quantification of SVIP. Second, *in vivo* blood samples are influenced by aging-related and systemic factors such as chronic neuroinflammation and cerebrovascular pathology (Hansson et al., 2023), which may influence SVIP levels. Furthermore, technical variations in extracellular vesicles isolation protocols (e.g., centrifugation parameters and surface marker enrichment methods) could also affect SVIP quantification (Vandendriessche et al., 2022; Tian et al., 2023). Despite these discrepancies, both lines of evidence suggest that SVIP may play a biologically relevant role in MCI-related processes. Future studies should integrate single-vesicle analysis technologies to trace the cellular origin of plasma SVIP-positive extracellular vesicles and conduct longitudinal research to delineate their dynamic trajectory across disease stages related to cognitive impairment.

Previous studies have demonstrated that plasma Aβ_42_/Aβ_40_ reflects CSF Aβ_42_/Aβ_40_ abnormalities with reported AUC values ranging from 0.81 to 0.87 (Palmqvist et al., 2023). Plasma p-tau217 has shown particularly high accuracy in identifying Aβ-PET–positive individuals (AUC 0.92–0.93) (Ashton et al., 2024). GFAP has also been recognized as a valuable auxiliary biomarker for AD, especially in plasma-based detection; however, it lacks disease specificity and may be influenced by coexisting neuropathologies (Baiardi et al., 2022). In our study, plasma SVIP distinguished phenotypic aMCI from cognitively unimpaired individuals with an AUC of 0.836. Although a direct head-to-head comparison within the same cohort was not performed, this performance falls within the range reported for other established plasma biomarkers in early-stage populations. It is important to note that established plasma biomarkers primarily reflect amyloid deposition, tau phosphorylation, or astrocyte activation, representing relatively downstream events in the classical amyloid–tau cascade. In contrast, SVIP is mechanistically linked to autophagy regulation and intracellular protein homeostasis. Increasing evidence suggests that autophagy dysfunction may occur early in the disease course and may precede the formation of overt amyloid plaques (Lee, 2022). Therefore, SVIP may capture a complementary biological process relevant to early-stage cognitive decline.

**Figure 6:**
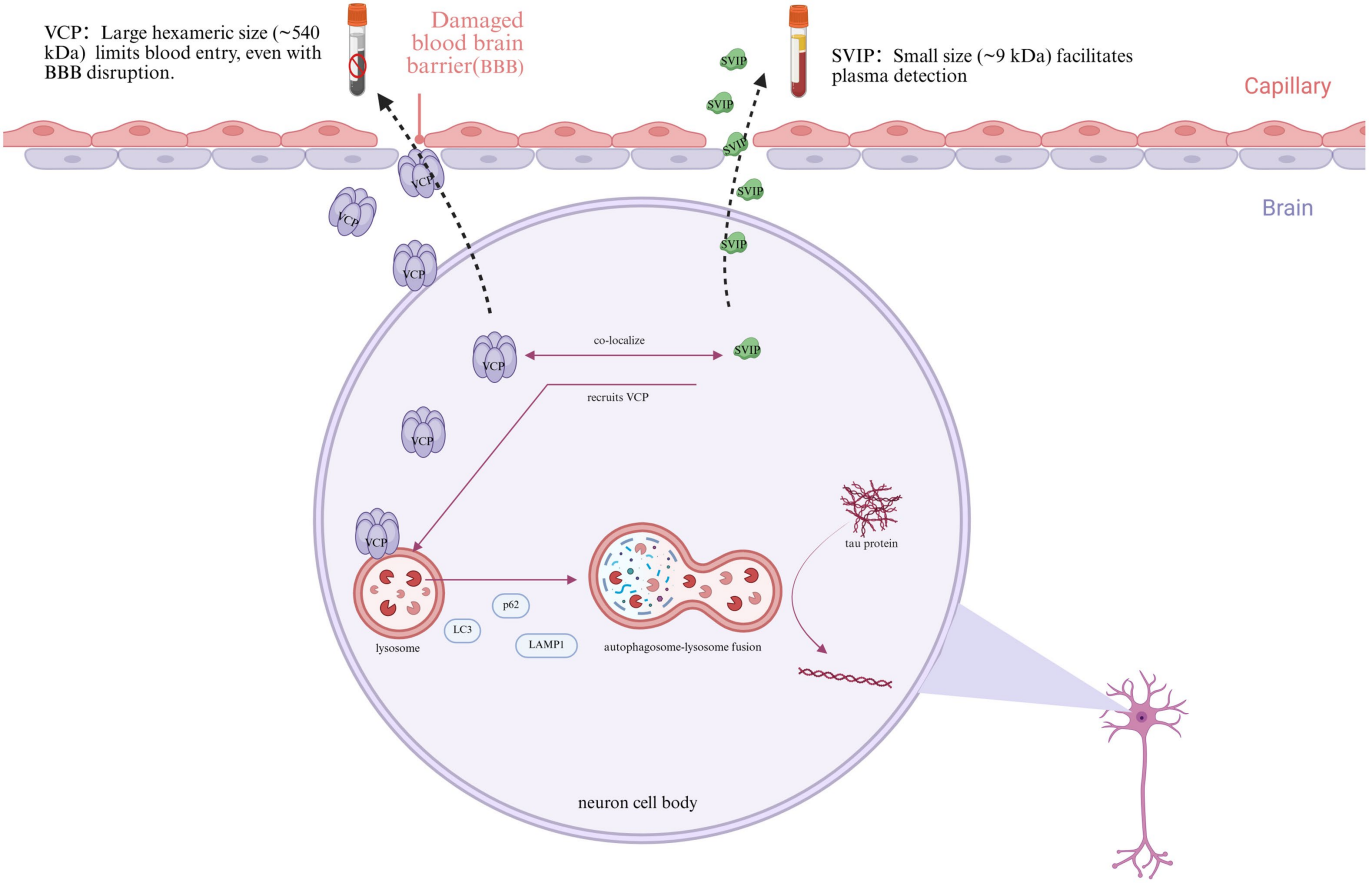
The potential process driving plasma SVIP and VCP changes. In neuron cell bodies, SVIP co-localizes with VCP and recruits it to the lysosomal membrane, facilitating autophagosome–lysosome fusion via LC3, p62, and LAMP1, a potentially important process in pathological tau degradation. Owing to its large hexameric size (~90 kDa per subunit), VCP may have limited passage across a disrupted blood–brain barrier (BBB), whereas the smaller SVIP (~9 kDa) could more readily be detected in the peripheral circulation. SVIP, small VCP-interacting protein; VCP, valosin-containing protein.

Unlike SVIP, although previous studies have reported decreased VCP expression in AD brain tissues, our study found no significant changes in its plasma levels at the phenotypic aMCI stage. We hypothesize that this discrepancy between central and peripheral expression may involve the following factors: Firstly, differences in BBB leakage may play a key role. As noted in previous studies (Haruwaka et al., 2019), molecules around 10 kDa can traverse a compromised BBB, whereas larger molecules (e.g., 40 kDa and 70 kDa) are largely restricted. VCP, with a molecular weight of approximately 90 kDa and typically forming a hexameric structure, may have limited capacity to enter the peripheral circulation even in the presence of BBB breakdown at the phenotypic aMCI stage. In contrast, SVIP, at only 9 kDa, is more likely to cross the BBB and appear in peripheral circulation (Tian et al., 2023; Lin et al., 2021), potentially accounting for its higher sensitivity and detectability as a plasma biomarker at the phenotypic aMCI stage. The potential process driving plasma SVIP and VCP changes is shown in Figure 6. Secondly, metabolic interference factors should also be considered. As with other plasma biomarkers such as ptau217, VCP levels in the blood may be modulated by systemic physiological factors, including hepatic and renal function, inflammation, and metabolic disturbances (Alkhalifa et al., 2023; Mielke et al., 2022; Syrjanen et al., 2022). These influences may diminish its neuronal specificity and diagnostic utility as a peripheral biomarker for phenotypic aMCI.

This study has several limitations. First, the relatively small sample size (84 participants) and single-center design may limit the statistical power and generalizability of the findings. We aim to increase the sample size and include multi-center samples in future studies. Second, the diagnosis of aMCI in this study was based on clinical evaluation and standardized neuropsychological assessments, without confirmation via AD-specific biomarkers such as amyloid PET or CSF. In addition, the exclusion of preclinical Alzheimer’s disease in the cognitively unimpaired group was based on plasma p-tau217 rather than combined amyloid and tau biomarkers; given that amyloid pathology may precede tau abnormalities, this may have resulted in the inclusion of individuals with preclinical AD. Although strict inclusion and exclusion criteria were applied to minimize potential confounders, residual heterogeneity cannot be entirely excluded. This heterogeneity may bias the observed associations toward the null, resulting in conservative estimates of the relationship between plasma SVIP levels and AD-related pathophysiology. Future studies incorporating biomarker-defined cohorts, including both amyloid-*β* and tau biomarkers, are warranted to further validate the specificity of SVIP for AD and its potential diagnostic utility. Third, plasma SVIP and VCP levels were quantified using LC–MS–based signal intensities and reported as relative abundances in arbitrary units (a.u.). As semi-quantitative biomarkers, these values may not be directly comparable across different analytical platforms or laboratories. Future studies using standardized quantitative assays are required to improve cross-platform harmonization and enhance translational applicability. Fourth, plasma measurements alone cannot precisely determine the tissue origin of circulating SVIP. Although SVIP is highly expressed in the central nervous system and mechanistically linked to neuronal autophagy regulation through its interaction with VCP, similar to other blood-based biomarkers such as p-tau and Aβ, its plasma levels may also be influenced by systemic physiological factors, including hepatic and renal function, inflammation, and metabolic disturbances. Therefore, the observed reduction in plasma SVIP in phenotypic aMCI should be interpreted with caution. While our findings are consistent with impaired neuronal autophagy in early Alzheimer’s disease, further studies incorporating neuron-derived extracellular vesicle analysis, CSF validation, and longitudinal designs are needed to clarify whether plasma SVIP predominantly reflects central neurodegeneration or broader systemic processes. Finally, the causal relationship between plasma SVIP decline and autophagy disruption remains unproven. Although efforts were made to exclude known disease interference through strict inclusion and exclusion criteria, potential comorbidities or effects related to autophagy may still have influenced plasma biomarker levels and should be more carefully controlled and validated in future research.

In summary, this study shows that plasma SVIP may be a potential candidate blood-based biomarker for the phenotypic aMCI stage, whereas the diagnostic sensitivity of VCP may be limited due to its large molecular weight. Further large-scale clinical studies are warranted to validate these findings and explore the underlying mechanisms and hypotheses.

## Data Availability

The raw data supporting the conclusions of this article will be made available by the authors, without undue reservation.

## Supplementary material

SUPPLEMENTARY FIGURE S1 Flow diagram of study participant selection. Detailed classification of exclusion criteria is provided in Supplementary Table S1. *Plasma ptau217 was measured only in CU participants. CU, cognitively unimpaired; aMCI, amnestic mild cognitive impairment; SVIP, small VCP-interacting protein; VCP, valosin-containing protein.
