# Supplementary figures and images for: SVIP in plasma: a candidate blood-based biomarker for early detection of amnestic mild cognitive impairment
Source: Front Aging Neurosci. 2026 May 29;18:1781331. doi: 10.3389/fnagi.2026.1781331 (PMC13260444; doi:10.3389/fnagi.2026.1781331)

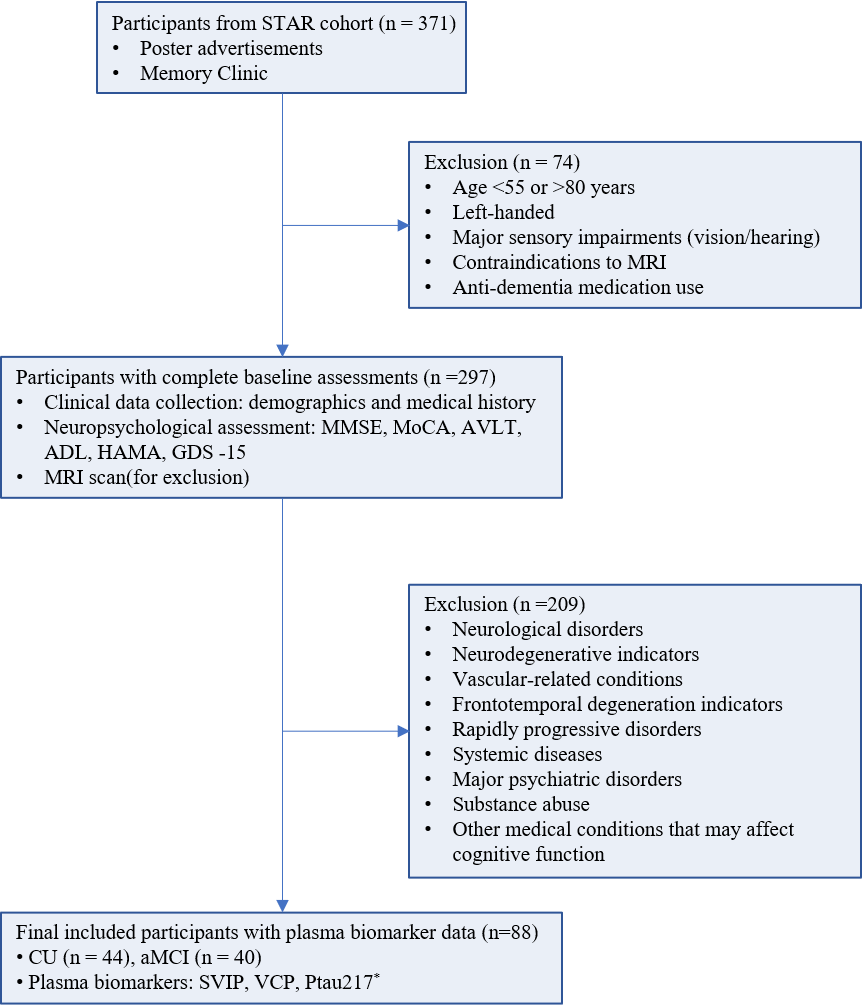

Supplement: SUPPLEMENTARY FIGURE S1 — Flow diagram of study participant selection. Detailed classification of exclusion criteria is provided in Supplementary Table S1. *Plasma ptau217 was measured only in CU participants. CU, cognitively unimpaired; aMCI, amnestic mild cognitive impairment; SVIP, small VCP-interacting protein; VCP, valosin-containing protein. [file Image_1.tif]
