# Supplementary material for: SVIP in plasma: a candidate blood-based biomarker for early detection of amnestic mild cognitive impairment
Source: Front Aging Neurosci. 2026 May 29;18:1781331. doi: 10.3389/fnagi.2026.1781331 (PMC13260444; doi:10.3389/fnagi.2026.1781331)
Supplement: Supplementary file 2 [file Data_Sheet_1.docx]

**TABLE S1** Detailed classification of exclusion criteria for study participants.

| Category | Specific conditions |
| --- | --- |
| Vascular-related conditions | Multiple vascular risk factors, extensive cerebrovascular lesions |
| Neurological disorders | Traumatic brain injury, intracranial tumors |
| Neurodegenerative indicators | Parkinsonism, visual hallucinations, REM sleep behavior disorder |
| Psychiatric disorders | Major depressive disorder, schizophrenia |
| Severe systemic diseases | Cardiovascular, hepatic, renal, and hematologic diseases |
| Substance abuse | Alcohol or drug dependence within the past 6 months |
| Other conditions | Other medical conditions that may affect cognitive function (e.g., hypothyroidism, gout) |

This table provides a systematic classification of disease-related exclusion criteria. Other exclusion factors are presented in the participant flow diagram (Supplementary Figure S1).

**TABLE S2A** Multiple linear regression analysis of SVIP and cognitive scores (MMSE and MoCA), adjusted for age and years of education.

| Variable | MMSE score | | | | MoCA score | | | |
| --- | --- | --- | --- | --- | --- | --- | --- | --- |
|  | *β* | 95% CI of *β* | *t*-Statistic | *p* | *β* | 95% CI of *β* | *t*-Statistic | *p* |
| SVIP (×10⁵ a.u.) | 0.18 | -0.03 to 0.38 | 1.70 | 0.094 | 0.98 | 0.53 to 1.42 | 4.38 | <0.001^*^ |
| Age (years) | -0.11 | -0.16 to -0.05 | -3.85 | <0.001^*^ | -0.24 | -0.36 to -0.12 | -3.99 | <0.001^*^ |
| Education (years) | 0.08 | 0.00 to 0.17 | 2.00 | 0.049^*^ | 0.43 | 0.25 to 0.61 | 4.76 | <0.001^*^ |

Data are presented as a regression coefficient (β) with 95% confidence interval (CI). Model adjusted for age and years of education. a.u., arbitrary units; CI, confidence interval; MMSE, Mini-Mental State Examination; MoCA, Montreal Cognitive Assessment. *^*^p < 0.05*.

**TABLE S2B** Multiple linear regression analysis of VCP and cognitive scores (MMSE and MoCA), adjusted for age and years of education.

| Variable | MMSE score | | | | MoCA score | | | |
| --- | --- | --- | --- | --- | --- | --- | --- | --- |
|  | *β* | 95% CI of *β* | *t*-Statistic | *p* | *β* | 95% CI of *β* | *t*-Statistic | *p* |
| VCP (×10⁶ a.u.) | -0.58 | -1.89 to 0.73 | -0.88 | 0.381 | 1.95 | -1.13 to 5.03 | 1.26 | 0.212 |
| Age (years) | -0.12 | -0.17 to -0.06 | -4.18 | <0.001^*^ | -0.27 | -0.40 to -0.14 | -4.08 | <0.001^*^ |
| Education (years) | 0.08 | -0.00 to 0.17 | 1.92 | 0.058 | 0.50 | 0.30 to 0.70 | 4.89 | <0.001^*^ |

Data are presented as a regression coefficient (β) with 95% confidence interval (CI). Model adjusted for age and years of education. a.u., arbitrary units; CI, confidence interval; MMSE, Mini-Mental State Examination; MoCA, Montreal Cognitive Assessment. *^*^p < 0.05*.
